# Rat Hepatocytes Weighted Gene Co-Expression Network Analysis Identifies Specific Modules and Hub Genes Related to Liver Regeneration after Partial Hepatectomy

**DOI:** 10.1371/journal.pone.0094868

**Published:** 2014-04-17

**Authors:** Yun Zhou, Jiucheng Xu, Yunqing Liu, Juntao Li, Cuifang Chang, Cunshuan Xu

**Affiliations:** 1 College of Life Science, Henan Normal University, Xinxiang, Henan, China; 2 Key Laboratory of Cell Differentiation and Regulation, Henan Normal University, Xinxiang, Henan, China; 3 College of Computer and Information Engineering, Henan Normal University, Xinxiang, Henan, China; 4 College of Mathematics and Information Science, Henan Normal University, Xinxiang, Henan, China; Schulze Center for Novel Therapeutics, Mayo Clinic, United States of America

## Abstract

The recovery of liver mass is mainly mediated by proliferation of hepatocytes after 2/3 partial hepatectomy (PH) in rats. Studying the gene expression profiles of hepatocytes after 2/3 PH will be helpful to investigate the molecular mechanisms of liver regeneration (LR). We report here the first application of weighted gene co-expression network analysis (WGCNA) to analyze the biological implications of gene expression changes associated with LR. WGCNA identifies 12 specific gene modules and some hub genes from hepatocytes genome-scale microarray data in rat LR. The results suggest that upregulated MCM5 may promote hepatocytes proliferation during LR; BCL3 may play an important role by activating or inhibiting NF-kB pathway; MAPK9 may play a permissible role in DNA replication by p38 MAPK inactivation in hepatocytes proliferation stage. Thus, WGCNA can provide novel insight into understanding the molecular mechanisms of LR.

## Introduction

The mammal liver has an impressive regenerative capability. Classical experiments in rats following partial hepatectomy (PH) have demonstrated that the liver can restore to its original size within 7–10 days. This regeneration capability can be utilized in clinical scenarios in which PH is used to resect liver tumors and in which living donor transplantation of liver is necessary in both the donor and recipient operations. Therefore, understanding the molecular mechanisms of LR is directly relevant to clinical problems. Prodigious ability to regenerate after PH has attracted the attentions of researchers for decades. However, at present, the molecular mechanisms of LR is still poorly understood [1].

Rat 2/3 PH is an established model for investigating the potential molecular mechanisms of LR. Many efforts have been made to study the molecular mechanisms of LR systemically and comprehensively with modern high-throughput biology techniques such as microarray, gene subtractive hybridization, series analysis of gene expression, and yeast two-hybrid system [2]. For example, Dransfeld et al. analyzed expression changes of the transport system-related genes in rat LR with oligonucleotide microarray containing 400 transcripts and identified 183 genes associated with LR following 2/3 PH [3]. Xu et al. examined the expression profiles of genes involved in physiological responses, cell metabolism, protein, enzymes, and biological active ingredients in LR utilizing cDNA microarray containing 551 transcripts, and found 133 known genes and 177 unknown genes related to LR following 2/3 PH [4], [5]. Yasuyuki et al. investigated gene expressions using cDNA microarray composed of 4,608 transcripts at 6, 12, 18, 24, 48, 72, and 168 h after 2/3 PH, and found 382 LR-associated genes, and also found that the gene expression profiles in 12 and 18 h, 48 and 72 h after PH were very similar [6]. However, the results of these studies mentioned above were mainly based on differential expression analysis. As a result, they usually generated a list of genes changed during LR but lacking biological functional connections among these genes [7]–[9].

It is well-known that LR induced by 2/3 PH is mainly mediated by hepatocytes proliferation. Hepatocyte replication underlies the restoration of liver mass in patients or liver donors following PH. Therefore, this study aims to analyze the intrinsic connections among the genes in hepatocytes during LR. LR is a complicated but well-orchestrated process with the synergistic work of a large number of genes [10]. Networks provide a straightforward representation of interactions among these genes. Intuitive network concepts (e.g. connectivity and module) have been found useful for analyzing complex interactions. Network analysis methods allow a more accurate reflection of underlying systems biology to be realized than traditional unidimensional molecular biology approaches [11]. Network-based systematic biology approaches [12] typically involve in the identification of groups of genes or network modules by microarray data analysis, whose expression levels are highly correlated across samples [13]–[15]. For example, He et al. identified novel dysfunctional modules and disease genes in congenital heart disease using a network-based approach [16]. Such holistic approaches have fully advantages over standard methods such as differential expression analysis.

Gene co-expression network-based approaches have become popular in analyzing microarray data, particularly for detecting functional gene modules [17], [18]. Dewey et al. identified ZIC2 as a novel transcription factor associated with co-expression modules common to developing and failing myocardium [15]. Zhang et al. predicted novel biomarkers for chronic lymphocytic leukemia using gene co-expression network analysis [19]. Puniya et al. integrated gene co-expression network analysis in the growth phase of mycobacterium tuberculosis and revealed new potential drug targets [20]. Childs et al. used gene co-expression network analysis as a source of functional annotation for rice genes [21].

In the study, we hypothesize that rat hepatocytes gene expression profiles in LR contain highly connected modifier genes and coordinate gene modules that will help to understand molecular mechanisms of LR and identify novel key genes related to LR. To test this hypothesis, the general framework for weighted gene co-expression analysis (WGCNA) is used to define the gene expression network topology of the LR hepatocytes. As co-expression modules may correspond to biological pathways [9], focusing on the analysis of modules will allow us to find novel molecular mechanisms. Using hierarchical average linkage clustering [22] based on topological overlap (TO), we identify 12 gene co-expression modules in the regenerating hepatocytes whose module preservation are significantly changed. Actually, quantitative assessment of module preservation in different phenotypes using both gene expression and network connectivity as summation [15], [23] provides a new avenue for understanding of molecular differences that distinguish functional processes in LR progression. Among these modules, one module involved in cell cycle regulation is found in PH hepatocytes, as a specific gene module, which is not found in normal hepatocytes. In addition, we also focus on highly connected intramodular hub genes which may be more biologically significant than that of the global network as well as several novel genes which may play important roles in LR. Although we do not find evidence for a global coordinated program of hepatocytes gene expression, our analysis reveals specific gene expression modules activated during LR and candidate hub genes for future experiment.

## Materials and Methods

### Materials

Healthy 12-week-old Sprague-Dawley (SD) rats, 230±20g, were obtained from the Experimental Animal Center of Henan Normal University. All the animal handling procedures were carried out in accordance with the current Animal Protection Law of China. The animal experiments were conducted in strict compliance with animal welfare regulations approved by Institutional Animal Care and Use Committee of Henan Normal University in China (Permit Number: SYXK2008-0105). All surgery was performed under amobarbital anesthesia, and all efforts were made to minimize suffering. In the experiment, a total of 114 rats were randomly divided into 19 groups, and 6 rats in each. Among these rats, 9 groups, total 54 rats for sham operation (SO), another 9 groups, total 54 rats for PH, and the rest 1 group, total 6 rats for the control. PH was performed on the rats according to the procedure originally described by Higgins et al. For SO, surgical operation of rats was done as did for the PH, but without liver lobes dissection. After that, the rats were bred and their regenerating livers were taken for isolating hepatocytes at 0, 2, 6, 12, 24, 30, 36, 72 and 168 h after PH. 0 h meant that the liver lobes were removed and the remaining liver was immediately used for hepatocytes isolation. The details of hepatocytes isolation, RNA extraction, microarray hybridization and RT-PCR validation were described in our earlier published paper [24].

### Real microarray dataset

In this study, we measured gene expression profiles of isolated hepatocytes from 2 h to 168 h after PH and SO with Rat Genome 230 2.0 array. Each sample corresponding to one time point was hybridized onto one array. The experiment was repeated 3 times for each time point. In total, 10 time points were measured and 0 h was used control group. After careful quality control analyses of each chip, the data were analyzed with Affymetrix GCOS 2.0 software using Affymetrix default analysis settings and global scaling as normalization method. The trimmed mean target intensity of each array was arbitrarily set to 500. When multiple probe sets were mapped to the same gene UniGene ID, the average expression vector was computed and used. As a result, 13925 known genes and 10693 unknown genes were mapped into 31099 probe sets of each microarray. For each gene, the ratio values of individual microarray signal vaules relative to the signal vaules of control group at each time point were used in this study. The raw and processed microarray data are available in the NCBI GEO database (accession number:GSE55434). From 13925 known genes, we selected 6995 most expression varied genes (t-test p-value<0.05) between PH group and SO group for further network analysis.

### Hepatocytes weighted gene co-expression network construction

The general framework for weighted gene co-expression analysis (WGCNA) is a systems biology method for describing the correlation patterns among genes and finding modules of highly correlated genes across microarray samples. This study uses WGCNA to construct rat hepatocytes gene co-expression network from microarray gene expression data. Firstly, we begin by calculating the Pearson correlations for all pairs of genes in the network. Because microarray data can be noisy and the number of samples is often small, the Pearson's correlation matrix for each co-expression network is transformed into a matrix of connection strengths using a power function(
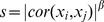
), here we choose 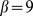 in accordance with the Scale-free Topology Criterion [14]. This step effectively serves to emphasize strong correlations and punish weak correlations on an exponential scale. These weighted correlations, in turn, represent the connection strengths between genes in the network. Then, these connection strengths are used to calculate the topological overlap (TO) as follows [25]. 
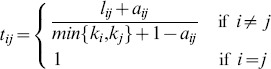
(1)


Where 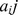 denotes the pairwise adjacency (connection strengh). 
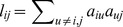
, 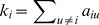. This step considers not only the correlation of the two genes, but also the degree of their shared neighbors across the whole network. R scripts for generating the WGCNA results in this study is available on line as Supporting Information files.

### Hepatocyte gene co-expression modules detection

Because gene modules may correspond to biological pathways, focusing on the analysis of modules amounts to a biologically meaningful data reduction scheme. In this study, hierarchical average linkage clustering based on TO is performed to identify gene co-expression modules, that is, groups of genes with similar patterns of expression across experimental samples. Since the module identification is computationally expensive, only 3600 most connected genes are considered for module detection. Because module genes tend to have high connectivity, this step does not lead to a big loss in information. We conduct the network module identification procedure [26] in a blockwise manner with the same parameter setting for both networks. To summarize the scaled gene expression profiles for the identified modules, we use the first singular vector (module eigengene, ME), which is equivalent to the first principle component and explains the largest proportion of variance of the module genes. We then use the MEs in a procedure to reassign genes to the modules that maximizes the module memberships [27]. To avoid capturing weak associations, genes with kME<0.3 for all of the MEs are assigned to none of them.

### Module preservation statistics

Module preservation statistics measures how well the modules of the reference network are preserved in the test network. Module preservation statistics during LR progression can be biologically meaningful (e.g., reflecting specific LR modules). Because preservation statistics measures different aspects of module preservation, the results may not always agree with each other. These measures can be classified into two categories: density based preservation statistics summarized by 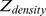 and connectivity based preservation statistics summarized by 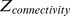 [28]. Density based preservation statistics can be used to determine whether module nodes remain highly connected in the test network. Connectivity based preservation statistics can be used to determine whether the connectivity pattern between nodes in the reference network is similar to that in the test network. We find that it is useful to aggregate different module preservation statistics into composite preservation statistics. Composite preservation statistics also facilitate a fast evaluation of many modules in multiple networks. Summarized composite statistics, 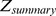, is defined as follows. 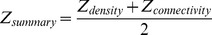
(2)


Many simulations [16], [28] have suggested the following thresholds for 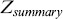: if 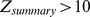, there is a strong evidence that the module is preserved; if 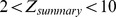, there is a weak evidence that module is preservation; if 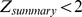, there is no evidence that the module is preserved.

### Gene significance (GS) and module member (MM) definition

For each of the 162 samples (81 in PH, 81 in SO), relative mRNA transcript level is available. To measure gene significance (GS) for each gene, we define GS of a gene as mediated t-test of differential expression between PH group and SO group. This provides a measure of how strongly each gene in the module is associated with LR. Abstractly speaking, GS is any quantitative measure that specifies how biologically significant a gene is.

The module eigengene (ME) corresponds to the first principal component of a given module. It can be considered as the most representative gene expression in a module. The module membership (MM) measure is determined by correlating the expression profile of a gene i with the ME of its resident module: 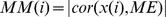 [14]. A MM correlation close to 1 means the gene is a member of the module since we define modules as sets of positively correlated genes. A correlation value close to 0 means that the gene is a member of none of modules.

### Hub genes detection

Highly connected intramodular hub genes may be more biologically significant than hub genes in global network. Thus, one of the goals of network analysis is to relate the measure of gene significance to module eigengene connectivity. We relate gene connectivity to gene significance. Gene connectivity is a measure of a gene's connection strength to other genes in the whole network. In general, connectivity is a more reliable measure than the t-test for differential expression. In practice, a combination of connectivity and differential expression should be used to select interesting genes. The MM measure is highly correlated with intramodular connectivity [14]. The intramodular connectivity may be interpreted as a measure of module membership. To identify hub genes for the network, we consider MM primarily and gene significance secondarily.

## Results

### Co-expression modules identified from PH and SO networks

**Figure 1:**
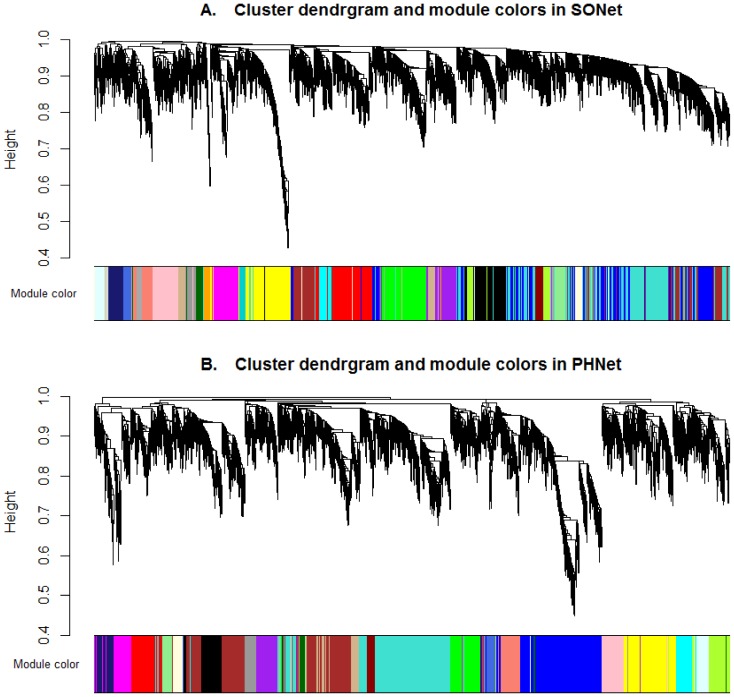
Identification of gene co-expression modules in SONet and PHNet. Hierarchical average linkage clustering was applied to gene-gene adjacencies, which were defined on the basis of TO. Dynamic tree cut algorithm was applied to the dendrogram for module identification, and genes in the same branch could be assigned to different modules. The analysis identified 29 modules (A) and 23 modules (B) represented by different colors on the horizontal bar.

Genes with expression levels that are highly correlated are biologically interesting, since they imply common regulatory mechanisms or participate in similar biological processes. We set out to investigate the hepatocytes transcriptome during rat LR and construct gene co-expression networks by applying WGCNA. This study was based on our real experiment data, which contains gene expression from 81 samples at 9 time points in SO group and PH group, respectively. For this study, we restricted the analysis to the 6995 known genes with high variation between PH group and SO group (t-test p-value<0.05). Firstly, the absolute value of the Pearson correlation between expression profiles of all pairs of genes was calculated. Then the Pearson correlation measure was transformed into a connection strength measure by using a power function (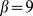). The connectivity measure for each gene was the sum of the connection strengths between that gene and all the other genes in the network. Gene expression networks, like virtually all types of biological networks, exhibited an approximate scale free topology. Two co-expression networks, SONet for SO group and PHNet for PH group, were constructed by TO for 3600 most connected genes (Figure 1).

To group genes with coherent expression profiles into modules, we used hierarchical average linkage clustering (see Materials and methods for details). Dynamic tree cut algorithm [26] was used to detect the modules (deep split  = 2, cut height = 0.995, other parameters were default values). As a result, we identified 29 modules in SONet and 23 modules in PHNet respectively (see table S1).

### Specific modules related to LR

**Figure 2:**
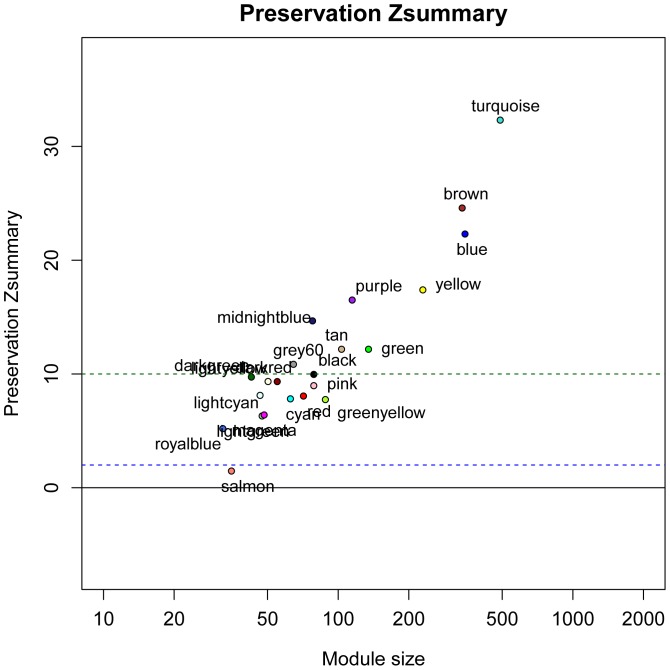
Composite preservation statistics of PHNet modules in SONet. The summary statistic 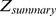 (y-axis) as a function of the module size, Each point represents a module, labeled by color. The blue and green horizontal lines show the thresholds of 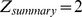 and 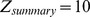, respectively. 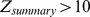 shows a strong evidence of preservation and 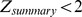 shows no evidence of preservations. 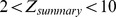 shows a weak evidence of preservation.

To identify LR-related gene modules, we assessed preservation of modules between two gene co-expression networks, SONet and PHNet. We adopted a previous measure of intramodular connectivity preservation [28]. We found that for PHNet, turquoise, blue, yellow, brown, purple, green, tan midnightblue, grey60 and black 10 modules were well preserved (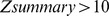) in SONet, it showed that these modules were housekeeping modules for keeping hepatocytes living. Nevertheless, Royalblue, lightgreen, magenta, greenyellow, cyan, lightcyan, red, pink, darkred, lightyellow and darkgreen module in PHNet were weak preserved in SONet (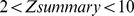), meaning that these modules had a significant change in intramodular connectivity in rat LR. Specially, salmon module (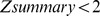) in PHNet was not found in SONet, suggesting that this module was a specific module in rat LR (Figure 2).

### Gene significance and hub genes

**Figure 3:**
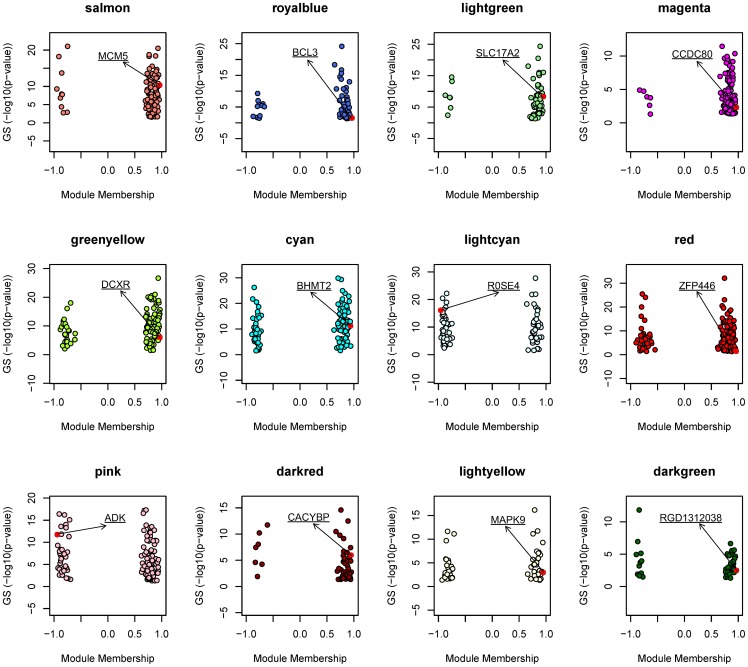
Correlation of Connectivity on the x-axis with gene significance on the y-axis and identification of hub genes based on high MM. The color represents the module and the dot represents the gene in the module. Red square represents hub gene in each module with highest connectivity. Hub gene symbol is underlined.

One of the goals in our network analysis is to relate the measure of differential expression to module connectivity. MM reflects the node importance in the network and GS reflects differential degree of expression of a single gene under different conditions. We related MM to GS for the genes in each module. Figure 3 showed MM vs GS for each differential module in PHNet. In general, a positive correlation could be expected between MM and GS. But, in this study, we did not observe a positive correlation between MM and GS. There are two possible causes. One is that only high connected genes are assigned to corresponding modules and genes with kME<0.3 for all of the MEs are assigned to none of them. The other is that high differential expressed genes do not necessarily have high intramodular connectivity during LR. We selected genes with maximum connectivity in each module as candidate LR-related genes, since they would be centrally located in the networks.

Identification of hub genes in PHNet modules involved relating MM on the x-axis to GS the y-axis (Figure 3). Because correlation between MM and GS was not strong, we selected novel hub genes based on high MM. MCM5 (MM.salmon = 0.96, p-value = 4.50E-11), BCL3 (MM.royalblue = 0.97, p-value = 0.029532), SLC17A2 (MM.lightgreen = 0.95, p-value = 4.19E-09), CCDC80 (MM.magenta = 0.96, p-value = 0.004953), DCXR (MM.greenyellow = 0.97, p-value = 9.83E-07), BHMT2 (MM.cyan = 0.94, p-value = 5.82E-12), ROSE4 (MM.lightcyan = -0.96, p-value =  8.09E-17), ZFP446 (MM.red = 0.96, p-value = 0.0334), ADK (MM.pink = -0.94, p-value = 1.72E-12), CACYBP (MM.darked = 0.95, p-value = 1.06E-06), MAPK9 (MM.lightyellow = 0.94, p-value =  0.001059) and RGD1312038 (MM.darkgreen = 0.97, p-value =  0.046652) were identified as highly connected hub genes in respective module.

### Gene functional annotations

To examine the relevance of distribution of the module genes with their biological roles in LR, functional annotations of the gene sets and modules were performed on the basis of their gene composition using DAVID software (http://david.abcc.ncifcrf.gov/). We further classified each module genes according to the gene classification based on functional categories. Top-ranked functional annotations enriched in LR-related modules were shown in Table 1. Gene functional analysis showed that salmon module was involved in similar biological activities, such as cell cycle, DNA metabolic process, mRNA transport, response to DNA damage stimulus, M phase of meiotic cell cycle et al. These biological activities were closely associated with cell cycle regulation of LR. So we called salmon module as cell cycle regulation module. Royalblue module was involved in positive regulation of anti-apoptosis and response to organic substance, peptide hormone stimulus, and endogenous stimulus. A detailed functional enrichment of GO annotations in these modules was provided in the table S2, and all GO terms mentioned in this section were highlighted in yellow background to facilitate search.

**Table 1:** Top-ranked functional annotations enriched in LR-related modules.

Module	Category	GOID	Term	P-Value
salmon	biological process	GO:0007049	cell cycle	2.24E-02
	biological process	GO:0022403	cell cycle phase	2.53E-02
	biological process	GO:0006259	DNA metabolic process	2.64E-02
royalblue	biological process	GO:0010033	response to organic substance	5.50E-04
	biological process	GO:0043434	response to peptide hormone stimulus	6.90E-04
	biological process	GO:0009719	response to endogenous stimulus	2.12E-03
	biological process	GO:0045768	positive regulation of anti-apoptosis	3.63E-03
lightgreen	biological process	GO:0045859	regulation of protein kinase activity	.30E-02
	biological process	GO:0043549	regulation of kinase activity	4.87E-02
magenta	biological process	GO:0030199	collagen fibril organization	3.87E-07
	biological process	GO:0001501	skeletal system development	1.71E-05
	biological process	GO:0030198	extracellular matrix organization	3.69E-05
greenyellow	biological process	GO:0016054	organic acid catabolic process	4.86E-07
	biological process	GO:0046395	carboxylic acid catabolic process	4.86E-07
	biological process	GO:0016054	organic acid catabolic process	4.86E-07
cyan	biological process	GO:0044271	nitrogen compound biosynthetic process	1.11E-04
	biological process	GO:0006575	cellular amino acid derivative metabolic process	4.14E-04
	biological process	GO:0008610	lipid biosynthetic process	5.64E-03
lightcyan	biological process	GO:0000375	RNA splicing, via transesterification reactions	3.65E-04
	biological process	GO:0000377	RNA splicing, via transesterification reactions with bulged adenosine as nucleophile	3.65E-04
	biological process	GO:0000398	nuclear mRNA splicing, via spliceosome	3.65E-04
red	biological process	GO:0042254	ribosome biogenesis	3.84E-07
	biological process	GO:0022613	ribonucleoprotein complex biogenesis	7.03E-06
pink	biological process	GO:0051297	centrosome organization	9.52E-03
	biological process	GO:0031023	microtubule organizing center organization	1.21E-02
	biological process	GO:0051603	proteolysis involved in cellular protein catabolic process	1.48E-02
darkred	biological process	GO:0006508	proteolysis	3.37E-03
	biological process	GO:0030163	protein catabolic process	4.58E-03
lightyellow	biological process	GO:0006605	protein targeting	1.01E-03
	biological process	GO:0006886	intracellular protein transport	1.48E-03
	biological process	GO:0034613	cellular protein localization	2.38E-03
darkgreen	biological process	GO:0012502	induction of programmed cell death	8.64E-02
	biological process	GO:0006917	induction of apoptosis	8.64E-02

All P-values from the Fishers Exact Test were Bonferroni-corrected.

### Pathway enrichment analysis

Pathway Studio is a pathway analysis tool supplied with RESNET mammal database. Pathway Studio harvests latest information from deposited literature in PubMed and other public sources. The software also uses a number of public and commercial databases such as KEGG (http://www.genome.jp/kegg/). We selected Ariadne Cell Process Pathways, Cell Signaling Pathways, Metabolic Pathways and Receptor Signaling Pathways for each module pathway enrichment analysis. It was found that cell cycle regulation (p-value = 1.23E-06) was top enriched in salmon module, IL6R->STAT signaling (p-value = 3.60E-04) was top enriched in royalbule module. TRRAP/Tip60 chromating remodeling (p-value = 8.18E-04) was top enriched in lightgreen module, ROS metabolism (p-value = 7.22E-04) was top enriched in magenta module, omega-3-fatty acid metabolism (p-value = 9.95E-06) was top enriched in greenyellow module, adipocytokine signaling (p-value = 9.12E-05) was top enriched in cyan module, tryptophan metabolism (p-value = 1.20E-02) was top enriched in lightcyan module, angiotensinR->STAT signaling (p-value = 7.51E-04) was top enriched in red module, notch->LEF1 signaling (p-value = 9.94E-04) was top enriched in pink module, FGFR1->STAT signaling (p-value = 1.05E-02) was top enriched in darkred module, frizzledR->JUN/PAX2 signaling (p-value = 2.30E-02) was top enriched in lightyellow module, irinotecan metabolism (p-value = 2.08E-05) was top enriched in darkgreen module. Top-ranked pathways enriched in LR-related modules were shown in Table 2. A detailed pathway enrichment of these modules was provided in table S3, and all pathways mentioned in this section were highlighted in yellow background to facilitate search.

**Table 2:** Top enriched pathways in LR-related modules.

Module	Pathway name	Total entities	Overlap	P-value	Data source
salmon	cell cycle regulation	135	25	1.23E-06	Ariadne cell signaling pathways
	histone acethylation	33	10	2.29E-04	Ariadne cell process pathways
royalbule	IL6R->STAT signaling	8	2	3.60E-04	Ariadne receptor signaling pathways
lightgreen	TRRAP/Tip60 chromating remodeling	35	4	8.18E-04	Ariadne cell process pathways
	INO80 chromating remodeling	25	4	1.15E-03	Ariadne cell process pathways
magenta	ROS metabolism	43	3	7.22E-04	Ariadne metabolic pathways
	DDR1->NF-kB signaling	14	2	1.00E-02	Ariadne receptor signaling pathways
greenyellow	omega-3-fatty acid metabolism	107	12	9.95E-06	Ariadne metabolic Pathways
	adipocytokine signaling	52	17	8.47E-05	Ariadne cell signaling pathways
cyan	adipocytokine signaling	52	13	9.12E-05	Ariadne cell signaling pathways
lightcyan	tryptophan metabolism	112	5	1.20E-02	Ariadne metabolic pathways
	notch pathway	40	11	4.57E-02	Ariadne cell signaling pathways
red	angiotensinR->STAT signaling	7	2	7.51E-04	Ariadne receptor signaling pathways
pink	Notch->LEF1 signaling	7	2	9.94E-04	Ariadne receptor signaling pathways
darkred	FGFR1->STAT signaling	13	1	1.05E-02	Ariadne receptor signaling pathways
lightyellow	frizzledR->JUN/PAX2 signaling	15	2	2.30E-02	Ariadne receptor signaling pathways
darkgreen	irinotecan metabolism	11	4	2.08E-05	Ariadne metabolic pathways

Total Entities represents the number of genes in the pathway. Overlap represents the number of overlapping genes between the pathway and the module.

## Discussion

In order to analyze which gene modules and underlying molecular mechanisms might play a key role during LR, this study was designed to construct rat hepatocytes gene co-expression network and identify gene co-expression modules by transcriptome data of hepatocytes following PH in rats. Although many efforts have been made to study LR transcriptome using various methods such as differential expression analysis whose result is usually a list of genes, each of which is deemed significant in isolation. However, the functional abnormity of a single gene rarely leads to complete regulation of LR. A drawback of the standard differential expression analysis is that it ignores the strong correlation patterns between the genes. As a result, it focuses on the details and ignores the bigger picture.

In this study, WGCNA was used to construct rat hepatocytes gene co-expression network topology, which considers not only the correlation between two genes, but also the degree of their shared neighbors across the whole network. Hierarchical average linkage clustering based on TO was used to group genes with highly similar co-expression patterns into modules. This method identified 23 high correlated modules in PH group. Among these modules, 11 modules have no significant change in LR process, called housekeeping modules. Another 12 modules have significant change within module connectivity, called specific LR-related modules.

The most interested module is the salmon module, which is found only in rat LR but not in normal hepatocytes. Salmon module is mainly enriched in cell cycle progression such as cell cycle (GO:0007049), cell cycle phase (GO:0022403), DNA metabolic process (GO:0006259), cell cycle process (GO:0022402), mRNA transport (GO:0051028), response to DNA damage stimulus (GO:0006974), M phase of meiotic cell cycle (GO:0051327) etc which are deemed to be driven by growth factors and cytokines, such EGF, HGF etc [1]. It suggests that salmon module plays an important role in hepatocytes proliferation during LR. Although this is not an unexpected finding, as a set of the highly correlated genes, pathways enriched in the module should be more accurate than those of enriched in overall differentially expressed genes. Here we reported a novel hub gene, MCM5 (p-value =  4.50E-11) was up-regulated (fold change>3) in hepatocytes after PH in rats. As a member of minichromosome maintenance (MCM) family, MCM5 is evolutionarily conserved from yeast to human. This protein is essential for DNA replication [29]. The signal transducer and activator of transcription proteins are critical for the signal transduction of a multitude of cytokines and growth factors leading to the regulation of gene expression. Zeng et al. have also found that activating MCM5 expression during transcription elongation promoted regenerative proliferation of adult stem cells [30]. Xu et al. have reported that MCM5 was up-regulated in hepatic oval cells after PH in rats [31]. Thus, MCM5 may be a novel key gene regulating cell cycle. This can be a promising protein for future experimental research. Another interesting module is royalblue with strong preservation in proliferation stage. This module is mainly enriched in response to organic substance (GO:0010033), peptide hormone stimulus (GO:0043434), endogenous stimulus (0009719) and positive regulation of anti-apoptosis (GO:0045768). Its hub gene BCL3 (B cell leukemia-3), a nuclear member of the IkappaB (inhibitor of NF-kB) family, that regulates a wide range of biological processes, including cell survival, proliferation, differentiation, stress response, and death, as well as immunity and inflammation. Aberrant NF-kB pathway activity is known to be associated with LR [32]. So BCL3 may be a novel key gene related to LR by NF-kB pathway. Lightyellow module is also the one interesting module, which are mainly enriched in protein gargeting (GO:0006605), intracellular protein transport (GO:0006886) and cellular protein localization (GO:0034613). Its hub gene MAPK9, as a member of mitogen-activated protein kinases (MAPK) family, plays a permissible role in DNA replication by p38 MAPK inactivation during LR and is consistent with a role for p38 MAPK in the maintenance of hepatocyte cell cycle arrest in adult liver [33].

In addition, we have also found some other novel hub genes in the remaining 9 modules, such as SLC17A2, CCDC80, DCXR, BHMT2, R0SE4, ZFP446, ADK, CACYBP, and RGD1312038. The roles of these hub genes in LR have not been reported in the literature.

Various pathways are observed in the network. Cell cycle regulation, histone acethylation and pyrimidine metabolism are top enriched in salmon module. IL6R->STAT signaling is top enriched in royalbule module. IL-6 is crucial to liver regenerative responses and cannot be substituted by other endogenously produced cytokines. With no hepatectomy, IL-6 does not provide a sufficient stimulus to promote hepatocyte proliferation [34]. Liver is a highly metabolic organ with a great potential of regeneration, LR is also triggered through metabolites of intracellular reactive oxygen species (ROS) and anti-apoptotic mechanisms [35]. Omega-3 polyunsaturated fatty acids enriched in greenyellow module may prevent acute liver failure and promote liver regeneration after 90% hepatectomy in rats [36]. Adipocytokine signaling enriched in cyan module is also interesting. Adiponectin, an important adipocytokine produced by fat cells, plays important roles in energy homeostasis. It binds to at least two receptors, known as AdipoR1 and AdipoR2, both of which are expressed in the liver. Shu and colleagues studied LR in a PH model in an adiponectin-knockout background. They found that LR was impaired in adiponectin-knockout mice, as illustrated by smaller regenerated livers that also exhibited decreased hepatocytes proliferation [37]. Notch pathway enriched in pink module is important for cellular differentiation and proliferation [38], but their roles in LR after PH are unclear. FGFR1 is a critical protein of FGFR1 pathway enriched in darkred module. Huang et al. confirmed that livers of transgenic FGFR1 mice exhibited accelerated regeneration after PH, meanwhile the persistent activity of ectopic FGFR1 in hepatocytes was a strong promoter of hepatocellular carcinoma by driving cell proliferation at early stages and promoting neo-angiogenesis at late stages of progression [39]. How to regulate LR by FGFR1->STAT signaling is unclear. In addition, the roles of frizzledR ->JUN/PAX2 signaling enriched in lightyellow and irinotecan metabolism enriched in darkgreen module in rat LR still need to be confirmed.

The LR after 2/3 PH is a characteristics model with distinct stages. It has been well documented that DNA replication of hepatocytes begins at post-PH 12h in rats, and the first wave of DNA synthesis occurs at 24 h, with a smaller peak at 36-66 h. The process of LR is generally completed within 7 days after PH. Therefore, the process of LR can be divided into 3 stages: priming stage (2-6 h): hepatocytes are activated and G0/G1 transition occurs; proliferative stage (6-72 h): cell proliferation take places; termination stage: (72-168 h): liver regeneration terminates. In order to determine stages during which these pathways and hub genes play a key role, we reconstructed 3 gene co-expression networks across corresponding samples using similar method. Then we calculated preservation statistics of these modules in respective network, see Table 3. As it can be seen that salmon module is formed in proliferation stage and its preservation become weak in termination stage. Approximate 20% genes of cell cycle regulation and 30% genes of histone acethylation are enriched in salmon module. It suggests that salmon module play an important role in regulating hepatocyte proliferation. Another interesting module we observed is royalblue module, which is also strongly preserved in proliferation stage only. IL6R->STAT signaling enriched in the module has been demonstrated its role in rat LR [34]. Approximate 10% genes of omega-3-fatty metabolism and 33% genes of adipocytokine signaling enriched in greenyellow module suggest that this module play an important role during overall LR, because this module is preserved strongly during all three stages of LR. Surprisingly, 25% genes of adipocytokine signaling are also significantly enriched in cyan module which is also preserved strongly in three stages. It suggests that expression patterns of the two modules are different and there may be some regulation relationships between the two modules. In addition, lightcyan and darkgreen module are also interesting. 27.5% genes of notch pathway are enriched in lightcyan module and 36% genes of irinotecan metabolism are enriched in darkgreen module. Lightcyan module appears in priming and proliferation stages, but darkgreen module appears in proliferation and termination stages.

**Table 3:** Module preservation statistics (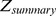) in distinct stages of LR after 2/3 PH.

Stage	priming (2-6h)	proliferation (6-72h)	termination (72-168h)
salmon	-0.65	19	8.6
royalbule	9	12	7.3
lightgreen	7.2	8.9	7.8
magenta	-0.88	25	18
greenyellow	11	12	17
cyan	21	13	12
lightcyan	12	15	9
red	23	22	8.8
pink	-0.09	21	17
darkred	11	20	11
lightyellow	8.7	13	7.9
darkgreen	9.5	15	11

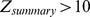
 represents that the module is strongly preserved in corresponding stage.

**Figure 4:**
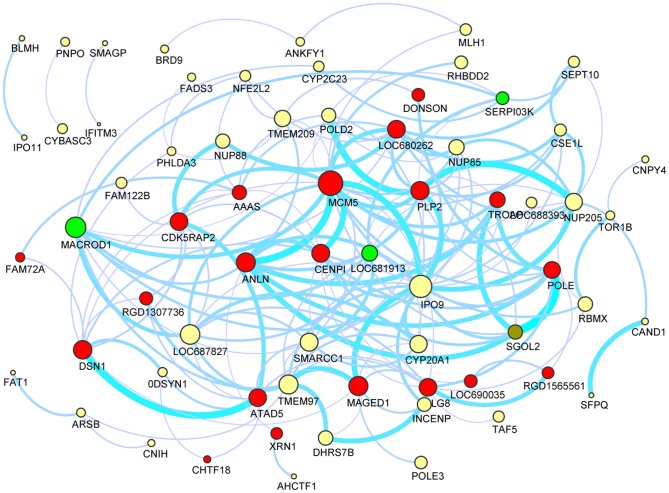
Predicated gene interactions by coexpression pattern in salmon module. For clarity, Only the edges corresponding to connection strenth > 0.3 were shown. The network was visualized using Cytoscape 3.0 software. Upregulated genes, downregulated genes, up/down-regulated genes and other genes were colored red, green, olive and yellow respectively. The node size is proportional to the node connectivity. The edge width is proportional to the connection strength between the two nodes. The Excel file of the map can be found in Table S4.

We also investigated the co-expression network of salmon module (Figure 4, the Excel file of the map was provided in table S4), representing the subset of genes consistently co-expressed in hepatocytes proliferation stage. We found several strong interactions, such as MCM5 interacting with ANLN, CENPI and IPO9 etc. We infer that it is this subset of genes that lead to hepatocytes proliferation and regulate cell cycle.

Although we have identified some specific modules in rat LR through WGCNA and confirmed the possible roles of these modules in LR through gene set enrichment analysis, whether some other pathways are also involved in LR cannot be excluded.

This is the first time that WGCNA is used to analyze the transcriptome information of rat hepatocytes in LR. Our results show that WGCNA provides considerable clues for further experiment by identifying critical gene modules and hub genes in LR. This study highlights the unique capability of WGCNA in prediction of novel undiscovered genes.

## Supporting Information

Table S1 **Module composition based WGCNA in PH group and SO group.**
(XLSX)

Table S2 **Detailed functional enrichment of GO annotations in LR-related modules.**
(XLSX)

Table S3 **Detailed pathways enriched in LR-related modules.**
(XLSX)

Table S4 **The gene interactions in the salmon module.**
(XLSX)

R Script S1 **R code used for generating the WGCNA results.**
(R) Click here for additional data file.
